# Disrupting the SKN-1 homeostat: mechanistic insights and phenotypic outcomes

**DOI:** 10.3389/fragi.2024.1369740

**Published:** 2024-03-04

**Authors:** Chris D. Turner, Carmen M. Ramos, Sean P. Curran

**Affiliations:** ^1^ Leonard Davis School of Gerontology, University of Southern California, Los Angeles, CA, United States; ^2^ Dornsife College of Letters, Arts, and Sciences, Department of Molecular and Computational Biology, University of Southern California, Los Angeles, CA, United States

**Keywords:** SKN-1, *C. elegans*, Nrf2, aging, cytoprotection, genetics, physiology, metabolism

## Abstract

The mechanisms that govern maintenance of cellular homeostasis are crucial to the lifespan and healthspan of all living systems. As an organism ages, there is a gradual decline in cellular homeostasis that leads to senescence and death. As an organism lives into advanced age, the cells within will attempt to abate age-related decline by enhancing the activity of cellular stress pathways. The regulation of cellular stress responses by transcription factors SKN-1/Nrf2 is a well characterized pathway in which cellular stress, particularly xenobiotic stress, is abated by SKN-1/Nrf2-mediated transcriptional activation of the Phase II detoxification pathway. However, SKN-1/Nrf2 also regulates a multitude of other processes including development, pathogenic stress responses, proteostasis, and lipid metabolism. While this process is typically tightly regulated, constitutive activation of SKN-1/Nrf2 is detrimental to organismal health, this raises interesting questions surrounding the tradeoff between SKN-1/Nrf2 cryoprotection and cellular health and the ability of cells to deactivate stress response pathways post stress. Recent work has determined that transcriptional programs of SKN-1 can be redirected or suppressed to abate negative health outcomes of constitutive activation. Here we will detail the mechanisms by which SKN-1 is controlled, which are important for our understanding of SKN-1/Nrf2 cytoprotection across the lifespan.

## 1 SKN-1 is a multifunctional cytoprotective transcriptional regulator

**FIGURE 1:**
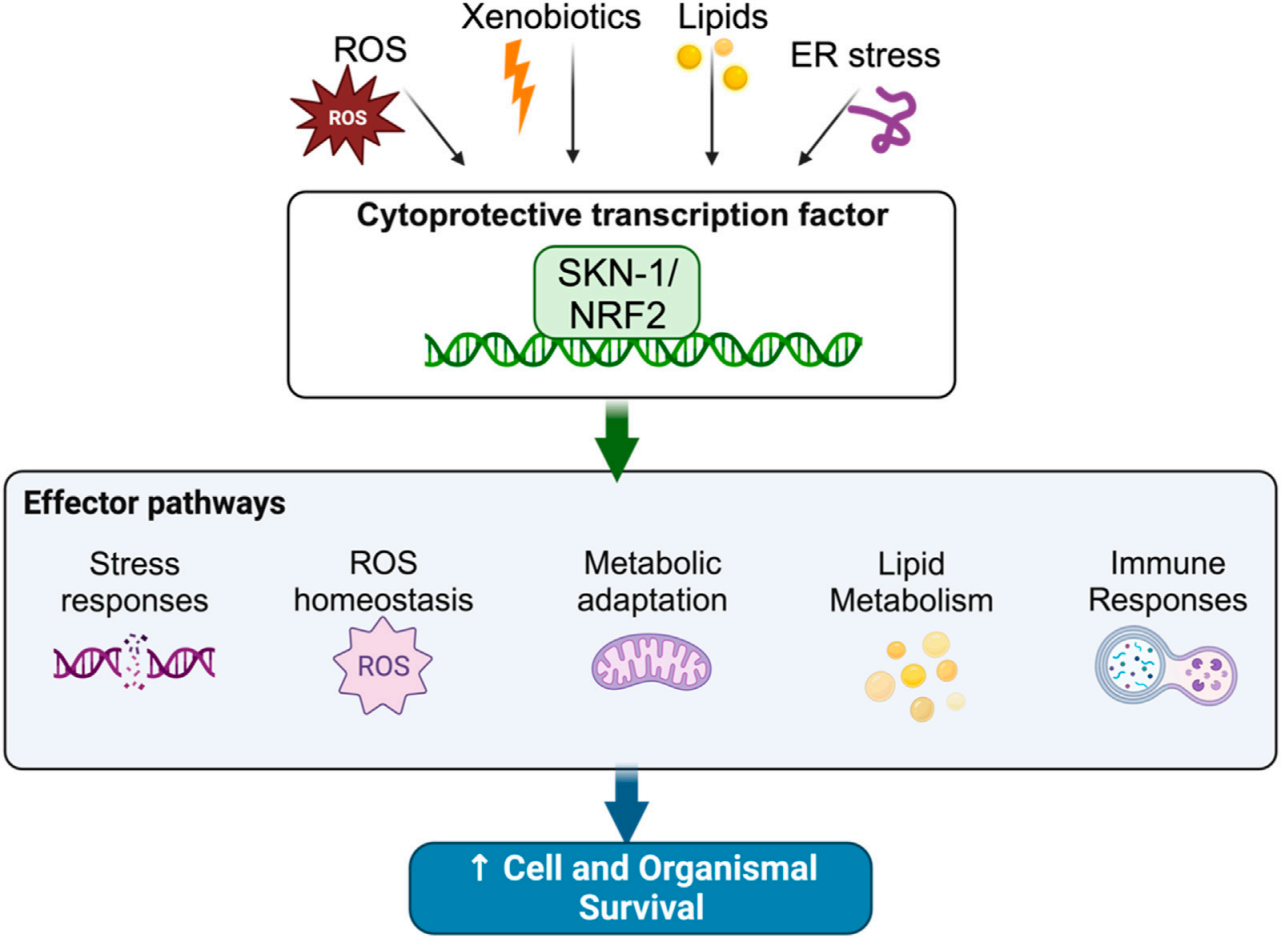
The SKN-1 homeostat maintains cellular balance in response to stress.

Homeostatic control mechanisms are crucial to the maintenance of cellular health and organismal longevity (Sykiotis and Bohmann, 2010; López-Otín et al., 2013; Blackwell et al., 2015; Torday, 2015; Cohen, 2016; Röder et al., 2016; Lemberg and Strisovsky, 2021; Ni and Buszczak, 2023). Indeed, several of the hallmarks of aging including genomic instability, mitochondrial dysfunction, and proteostatic disruption are all caused by the inability to maintain cellular homeostasis (Sykiotis and Bohmann, 2010; López-Otín et al., 2013; Blackwell et al., 2015; Torday, 2015; Cohen, 2016; Röder et al., 2016; Lemberg and Strisovsky, 2021; Ni and Buszczak, 2023). Xenobiotics and Reactive Oxygen Species (ROS) drive cellular instability by damaging cellular components; to respond to this, organisms have evolved stress tolerance pathways that can abate damage caused by xenotoxic stress (Sykiotis and Bohmann, 2008; Sykiotis and Bohmann, 2010; Kansanen et al., 2013; López-Otín et al., 2013; Blackwell et al., 2015; Torday, 2015; Cohen, 2016; Röder et al., 2016; Lemberg and Strisovsky, 2021; Ni and Buszczak, 2023). At the center of this homeostat, in worms and mammals, are the “Cap’n’Collar” (CnC) transcription factors skinhead-1 (SKN-1) and NF E2-Related Factor (Nrf2), respectively (Figure 1).

### 1.1 Structural comparisons of Nrf2 and SKN-1

Nrf2 is comprised of four major domains (Itoh et al., 1995). The DNA binding domain, which contains the CnC domain and Basic Region domain, followed by a basic leucine zipper domain (bZIP) that facilitates heterodimerization with Maf proteins to promote DNA binding (Itoh et al., 1995). Upstream of the DNA binding domain and ZIP region is the transcription activation DIDLID domain (Katoh et al., 2005). While SKN-1 is generally structurally diverged from its mammalian orthologue Nrf2, the CnC and Basic Region domains remain conserved (Blackwell et al., 1994; Carroll et al., 1997). Interestingly, SKN-1 lacks the leucine zipper domain that characterizes Nrf2 and other species of CnC transcription factors (Blackwell et al., 1994; Carroll et al., 1997). *In vitro* studies suggest that SKN-1 has adapted to bind DNA as a monomer at similar bZIP recognition sequences to Nrf2, via an enhanced Homeodomain Arm that interacts with the minor groove of the DNA helix where Maf proteins would bind in mammals (Blackwell et al., 1994; Carroll et al., 1997; Lo et al., 1998; Rupert et al., 1998; Kophengnavong et al., 1999). It is currently unclear if SKN-1 forms any dimers *in vivo*.

Nrf2 is part of a family that comprises three proteins each with distinct expression patterns, regulation, and gene targets (Sykiotis and Bohmann, 2010). Nrf2 is the most well characterized of these three proteins, it controls the expression of Phase II Detoxification genes that remove ROS-damaged molecules from the cell (Motohashi and Yamamoto, 2004). Nrf2 is negatively regulated by the cytoplasmic protein Kelch-like ECH associated protein 1 (Keap1) (Motohashi and Yamamoto, 2004). Keap1 binds to Nrf2 and promotes proteasomal degradation (Motohashi and Yamamoto, 2004). Upon electrophilic stress, cystine residues on Keap1 are reduced, weakening association with Nrf2 (Itoh et al., 2004; Motohashi and Yamamoto, 2004; Uruno and Motohashi, 2011). Once free of this negative regulation, Nrf2 localizes to the nucleus and binds at the Antioxidant Response Element (ARE), a recognition sequence in the promoters of detoxification response genes, which upregulates transcription at these loci (Itoh et al., 2004; Motohashi and Yamamoto, 2004; Uruno and Motohashi, 2011). Nrf1 is embedded into the membrane of the Endoplasmic Reticulum (ER) via an N-terminal transmembrane domain (Chan et al., 1993; Ruvkun and Lehrbach, 2023). During proteasome stress, Nrf1 is cleaved from the ER membrane, translocates into the nucleus, and upregulates proteasome subunits (Ruvkun and Lehrbach, 2023). The least well characterized Nrf family member is Nrf3 (Chevillard and Blank, 2011). Like Nrf1, Nrf3 also associates with the ER membrane, however, its methods of regulation are more complex, and its downstream targets are not well characterized (Chevillard and Blank, 2011).

Unlike mammals, *C. elegans* only have one orthologue to the Nrf family, SKN-1 (Blackwell et al., 2015). SKN-1 has three functional isoforms SKN-1A, SKN-1B, and SKN-1C (Blackwell et al., 2015). Remarkably the isoforms of SKN-1 have functional overlap with the different members of the Nrf family (Blackwell et al., 2015). SKN-1A is the only isoform with a transmembrane domain whereby it associates with the ER membrane (Ruvkun and Lehrbach, 2023). During proteasome stress, SKN-1 is cleaved from the ER and upregulates proteasome subunits (Ruvkun and Lehrbach, 2023). SKN-1C, like Nrf2, is thought to be primarily involved in the cytoprotective response against ROS (Blackwell et al., 2015). Lastly, SKN-1B is the less well understood SKN-1 isoform (Blackwell et al., 2015). GFP tagging of SKN-1 suggests that SKN-1B remains entirely localized to a pair of sensory neurons called ASI and contributes to longevity induced by caloric restriction (Bishop and Guarente, 2007). While SKN-1 has significantly diverged from the Nrf proteins in mammals in both expansion and DNA binding mode, SKN-1 still shares functional overlap with the mammalian Nrf transcription factors. The functional similarities between each isoform of SKN-1 and the different members of the Nrf family indicate that there is evolutionary conservation of CnC transcription factors and that *C. elegans* could be used as a model to understand Nrf biology.

### 1.2 SKN-1 roles in embryonic development

SKN-1 was initially described as a maternally deposited mRNA for a tissue specification factor (Bowerman et al., 1992; Bowerman et al., 1993). SKN-1 is required for the differentiation of the EMS blastomere into the MS and E cells, which will further differentiate into the pharyngeal and intestinal tissues, respectively (Bowerman et al., 1992; Bowerman et al., 1993). Genetic analysis of loss-of-function alleles of *skn-1* (*zu67* and *zu138*) demonstrated a failure of embryos to differentiate the EMS blastomere into the proper tissue types, instead differentiating into additional hypodermis and body wall muscle (Bowerman et al., 1992; Bowerman et al., 1993). SKN-1 is positioned at the top of the specification/differentiation cascade of the EMS blastomere (Maduro et al., 2001a). SKN-1 initiates this by binding to the genes *med-1* and *med-2,* which will bind other differentiation factors, determining which daughter cell will become the MS or E blastomere (Maduro et al., 2001a). Only *Nrf1* is required for development in mice, with *Nrf1* ^*−/−*^ mice having liver development abnormalities (Chen et al., 2003). However, Nrf1 in mice contributes to hepatocyte maturation by protecting from Tumor Necrosis Factor mediated apoptosis and maintaining redox balance and does not act as a specification factor in early embryogenesis like SKN-1 (Chen et al., 2003). While the requirement for SKN-1 in the development of the intestine is clear, what remains unknown is which isoforms of SKN-1 contribute to development and how. The *skn-1* loss of function alleles *zu67* and *zu138* ablate the function of all SKN-1 isoforms, making it difficult to determine which isoform(s) contribute to development. If we look to mammals as an example where Nrf1 is involved in development, it may mean that SKN-1A is responsible for development in the worm. Using genetic nulls for each isoform will inform which isoform is responsible for intestinal specification.

### 1.3 SKN-1 roles in oxidative stress response

Many of the postembryonic investigations done on SKN-1 have focused on its role in upregulating detoxifying pathways. Like mammals, *C. elegans* requires a robust mechanism for detoxifying xenobiotics and endogenous toxins. This process is broken into three phases (Xu et al., 2005; Lindblom and Dodd, 2006). Phase I detoxification occurs as cytochrome P450s and short chain dehydrogenases conjugate reactive groups onto toxic molecules increasing solubility and allowing further detoxifying metabolism to occur (Xu et al., 2005; Lindblom and Dodd, 2006). Phase II detoxification is comprised of enzymes that conjugate glutathione, a reducing agent, onto reactive groups (Xu et al., 2005; Lindblom and Dodd, 2006). These enzymes include Glutathione-S Transferases (GSTs) and UDP-glucosyl Transferases (UGTs) (Xu et al., 2005; Lindblom and Dodd, 2006). Finally, Phase III detoxification is comprised of transporters, including ATP-Binding Cassette (ABC) transporters (Xu et al., 2005; Lindblom and Dodd, 2006). These will move the fully solubilized and neutralized toxins out from the cytoplasm (Xu et al., 2005; Lindblom and Dodd, 2006). Microarray, RNA-seq, and ChIP-seq studies have determined that SKN-1 upregulates a bevy of Phase I, II, and III components as direct targets (Oliveira et al., 2009; Staab et al., 2013; Staab et al., 2014; Steinbaugh et al., 2015; Ramos and Curran, 2023; Turner et al., 2023).

Early work on the role of SKN-1 in the detoxifying response to ROS relied on GFP fusion expression arrays that tagged SKN-1 at the C-terminus, tagging each isoform simultaneously (An and Blackwell, 2003). These visualization experiments determined that under normal conditions SKN-1GFP accumulates within two sensory neurons at the head of the animals called ASI (An and Blackwell, 2003). Furthermore, when acutely exposed to sodium arsenite, SKN-1GFP accumulated in the intestinal cell nuclei, marking the intestine as a major site for SKN-1-mediated detoxification (An and Blackwell, 2003). In addition to visualization experiments, genetic analysis using the *skn-1* loss-of-function alleles *zu67* and *zu129* determined that without functional SKN-1, animals had diminished survival when exposed to acute oxidative stress (An and Blackwell, 2003). Furthermore, SKN-1 loss-of-function alleles display shortened lifespans in comparison to wildtype worms, establishing SKN-1 as both a stress response factor and a longevity maintenance factor (An and Blackwell, 2003). While SKN-1 is implicated in both stress adaptation and longevity, it is likely that these roles are deeply interconnected, given that in situations where SKN-1 is required for longevity, loss of SKN-1 not only abates longevity but also abates stress adaptation. Furthermore, in constitutively active *skn-1* mutants, where longevity is decreased, stress adaptation is improved (Paek et al., 2012; Blackwell et al., 2015).

It has long been appreciated that SKN-1 mediates the oxidative stress response via the intestine. However, it remains less clear what SKN-1 does in the ASI neuron pair. Bishop and Guarente used isoform specific RNAi to knockdown *skn-1b* gene products, and found that RNAi to *skn-1b* diminished the brightness of a SKN-1 activity reporter in the ASI neurons (Bishop and Guarente, 2007). Additionally, they found that SKN-1B was required for lifespan extension caused by caloric restriction, while SKN-1A/C had no effect on this phenotype (Bishop and Guarente, 2007). A recent investigation into cell non-autonomous roles of SKN-1 has given better insight into SKN-1 activity in the ASI neurons (Turner et al., 2023). Cell type-specific RNA-seq on the ASI neurons, in an activated SKN-1 background, showed an increase in typical SKN-1 targets including Phase II detoxification, components of the ubiquitin proteasome, and pathogen response genes (Turner et al., 2023). This introduces new hypotheses about SKN-1 in the ASI neurons. Either SKN-1B has remarkable overlap with SKN-1A/C targets and regulates all SKN-1 targets in the ASI neurons, or the other isoforms of SKN-1 are active in the ASI neurons. Supporting the hypothesis that other SKN-1 isoforms are active in the ASI neurons, a cell type expression construct expressing an activated allele of *skn-1c* in the ASI neurons resulted in the activation of the *gst-4p*:GFP SKN-1 activity reporter across multiple tissues (Turner et al., 2023). However, when activated, *skn-1c* was expressed in singular tissues and animals were assessed for survival from acute oxidative stress, none could achieve a robust level of survival associated with activated SKN-1 (Turner et al., 2023). Taken together, these data suggest that neuronal SKN-1 controls more than the longevity response to caloric restriction. The activation of SKN-1 transcriptional targets in the neurons can initiate a multi-tissue stress response (Turner et al., 2023). The initiation of the stress response may require one or more tissues or the activity of more than one SKN-1 isoform to produce a fully effective response to oxidative stress (Turner et al., 2023); delineation of the tissue-specific roles of each isoform is of critical importance.

### 1.4 SKN-1 roles in proteasome stress

While SKN-1C/Nrf2 manages the response to oxidative stress, the membrane-bound SKN-1A/Nrf1 contributes to the response to proteasomal stress (Glover-Cutter et al., 2013). Under conditions where the proteasome is functional, SKN-1A is typically stored within the ER lumen where it is N-linked Glycosylated at several sites (Glover-Cutter et al., 2013; Lehrbach and Ruvkun, 2016; Lehrbach et al., 2019; Yoshida et al., 2021). SKN-1A is extruded into the cytoplasm via the ER Associated Degradation pathway and degraded by the proteasome. During conditions where the proteasome is inhibited, SKN-1A is extruded into the cytoplasm but is not degraded by the proteasome (Lehrbach and Ruvkun, 2016; Lehrbach et al., 2019; Yoshida et al., 2021). Instead SKN-1A undergoes significant post-translational modification (Lehrbach and Ruvkun, 2016; Lehrbach et al., 2019; Yoshida et al., 2021). The protease DDI-1 interacts with SKN-1A and cleaves the transmembrane domain from SKN-1A (Lehrbach and Ruvkun, 2016; Lehrbach et al., 2019; Yoshida et al., 2021). Further modification is performed by the peptide N-glycanase PNG-1. PNG-1 deglycosylates SKN-1A at the residues that were glycosylated inside the ER lumen (Lehrbach and Ruvkun, 2016; Lehrbach et al., 2019; Yoshida et al., 2021). Interestingly, the N-glycosylation within the ER lumen and subsequent deglycosylation by PNG-1 catalyzes sequence editing on SKN-1A, whereby the N-glycosylated asparagine residues are converted to aspartic acid residues (Lehrbach and Ruvkun, 2016; Lehrbach et al., 2019; Yoshida et al., 2021). Both mutation analysis, mutating the asparagine residues to aspartic acid, and assays reducing the function of PNG-1 have demonstrated that the sequence editing of SKN-1A is required for it to upregulate target genes (Lehrbach and Ruvkun, 2016; Lehrbach et al., 2019; Yoshida et al., 2021). SKN-1A will upregulate proteasome subunits thus increasing proteasome function and alleviating acute proteasome stress (Lehrbach and Ruvkun, 2016; Lehrbach et al., 2019; Yoshida et al., 2021). Remarkably SKN-1C and SKN-1A share many regulators such as, ERK1/2, mTORC1, and OGT, and while evidence suggests that sequence editing on SKN-1A fine tunes it to proteasome subunit targets, it is unclear if SKN-1A and SKN-1C synergize or overlap with each other. While large strides have been made in defining the post-translational modification and subsequent activation of SKN-1A from the ER, further studies should focus on the role SKN-1A might play in metabolism and nutrient sensing given its regulation by these pathways, along with potential synergies with SKN-1C that is also implicated in metabolism regulation. Furthermore, advancements in tissue-specific expression will allow for tissue and cell type investigation into SKN-1A function.

### 1.5 SKN-1 roles in mitochondrial homeostasis

The impairment of mitochondrial homeostasis is a hallmark of aging and age-related pathologies. Healthy cells utilize autophagy of mitochondria organelles (mitophagy), that selectively degrade damaged mitochondria (Youle and Narendra, 2011). *C. elegans* requires a robust mitophagy pathway to promote longevity in canonically long-lived mutants, such as *daf-2* mutants (Palikaras et al., 2015), that disrupt insulin signaling. In *C. elegans,* mitophagy is primarily controlled by DCT-1 (Palikaras et al., 2015). DCT-1 is localized to the outer mitochondrial membrane where it co-localizes with autophagosomal protein LGG-1, inducing mitophagy (Palikaras et al., 2015). Loss of DCT-1 via RNAi knockdown causes a loss of mitochondrial network integrity and under nominal conditions, loss of DCT-1 does not shorten lifespan (Palikaras et al., 2015). Loss of DCT-1 activity results in SKN-1 activation, SKN-1 in turn upregulates mitophagy factors including DCT-1 (Palikaras et al., 2015). During dietary restriction, respiration rates increase, and ablation of mitochondrial electron transport chain suppresses the longevity increase in dietarily restricted worms (Bishop and Guarente, 2007). Correspondingly, loss-of-function alleles of *skn-1* subjected to dietary restriction, fail to increase mitochondrial respiration, and rescue of the *skn-1b* isoform in the ASI neurons facilitates increased respiration and longevity under dietary restriction (Bishop and Guarente, 2007). Recently, the natural product tomatidine was shown to upregulate mitophagy in both worms and human cell culture by inducing SKN-1/Nrf2 activity, improving healthspan in *C. elegans* (Fang et al., 2017). In addition, SKN-1 was previously shown to interact with the mitochondrial surface protein Phosphoglycerate Mutase 5 (PGAM-5) via *in vitro* co-immunoprecipitation assays (Paek et al., 2012). Activating mutations in *skn-1* weaken the interaction between PGAM-5 and SKN-1, suggesting that SKN-1 activation may release SKN-1 from associations with PGAM-5 at the mitochondrial outer membrane (Paek et al., 2012). These studies establish SKN-1 as a mediator of mitochondrial homeostasis. Additional work should focus on establishing a mechanism for SKN-1 association at the outer mitochondrial membrane and possible interaction with mitophagy factors as a sensor for mitochondrial network integrity. Further exploration of natural products and derivatives is warranted as enhancing mitophagy at advanced age may be a beneficial treatment for age related disease.

## 2 SKN-1 responses to diet and metabolism

### 2.1 Dietary impact on physiology

The influence of bacterial diets on the physiology of *C. elegans* extends across various life history traits, encompassing development, reproduction, healthspan, and longevity (Soukas et al., 2009; Hansen et al., 2013; Bansal et al., 2015; Stuhr and Curran, 2020). Numerous studies have elucidated diet-induced effects, correlating with observable differences in metabolic profiles, lipid homeostasis, and feeding behavior (Soukas et al., 2009; Xiao et al., 2015). In a study from Stuhr and Curran (2020), they demonstrated that distinct bacterial species isolated from the laboratory environment, which mirror those found in *C. elegans’* natural habitats, can selectively alter development, reproduction, and metabolism (Stuhr and Curran, 2020). Their analysis of Gene Ontology (GO) terms associated with unique genes differentially expressed in animals fed *Methylobacterium, Xanthomonas,* or *Sphingomonas* revealed distinct molecular signatures for each diet. When compared to age-matched animals fed the standard laboratory diet *E. coli*/OP50 B, significant alterations were identified, among the identified genes, many played roles in development (*sqt-2*, *mlt-7, mlt-10*), metabolism (*fat-5, fat-7*), reproduction (*col-71, ptr-1, lon-1*), and aging (downregulation of *daf-2* and *age-1*, and upregulation of DAF-16/FoxO target genes). Notably, each bacterial diet corresponded to a specific metabolic profile, and *C. elegans* reared on these bacteria exhibited variations in intracellular lipid distribution. Examination of gene expression related to lipid metabolism revealed that approximately one-third of the differentially expressed genes on each bacterial food source has the potential to impact a pleiotropic physiological response, highlighting the intricate interplay between diet and regulation of fundamental biological processes in *C. elegans.* The understanding of how specific dietary components affect both health and lifespan in *C. elegans* can contribute to explaining variations in aging rates and the severity of age-related diseases.

It is notable that SKN-1 serves as a mediator in translating dietary influences into physiological outcomes. While live bacteria are conventionally utilized as the primary source in *C. elegans* studies, studies have indicated host-pathogen defenses stemming from the pathogenic nature of *E. coli* (Garigan et al., 2002; Irazoqui et al., 2010; Kumar et al., 2019). Studies employing dead bacteria, either through UV-killing or bactericidal antibiotics, have demonstrated an extension in lifespan by mitigating bacterial proliferation within the pharynx and intestine (Garigan et al., 2002), along with distinct impacts on physiological metrics such as growth, lipid metabolism, and movement (Stuhr and Curran, 2023). Nhan *et al* (2019) examined that feeding *skn-1* gain-of-function mutant animals with dead bacteria resulted in healthier lipid metabolic outcomes compared to their counterparts fed live bacteria (Nhan et al., 2019). This intricate relationship between diet and physiological outcomes, influenced by specific bacterial species, underscores the relevance of SKN-1 in orchestrating diverse responses, providing a potential link to the regulation of fundamental biological processes across organisms.

### 2.2 Gene-diet interactions and SKN-1 coordination

Diet and genetics are pivotal in shaping the intricate web of metabolic regulation, healthspan, and lifespan. Studies have identified neuroendocrine signaling pathways, such as the neuromedin U receptor (NMUR), are important for sensing dietary cues from different food types and are involved in regulation of lifespan in a diet-dependent manner (Maier et al., 2010). Specifically, *nmur-1* mutants lived longer on the standard *E. coli*/OP50 B diet than on the *E. coli/*HT115 K-12 diet, illustrating a notable gene-diet interaction (Maier et al., 2010). Recent work has revealed insight into adaptive response mechanisms that organisms utilize to maintain physiological homeostasis when exposed to different diets (Pang and Curran, 2012; Pang and Curran, 2014). Work by Pang and Curran (2014) on mutants of *alh-6*, a proline metabolism gene, displayed a shortened lifespan on the standard OP50 diet, but not on the HT115 diet, their findings further revealed a mechanism by which diets will signal a mitochondrial adaptation through coordination of neuronal and metabolic tissues upon exposure to the *E. coli*/HT115 K-12 diet (Pang and Curran, 2014). Within this context, the transcription factor SKN-1 has emerged as a key player; from its activation in long-lived mutants to its intricate involvement in oxidative stress and detoxification pathways, offering potential avenues for further exploration in this intricate landscape of gene-diet interactions. While significant strides have been made in deciphering gene-diet interactions and understanding the impact of diverse bacterial diets on life history traits in *C. elegans*, the molecular underpinnings of these dietary responses remain a subject of ongoing investigation (Lynn and Curran, 2015).

### 2.3 Nutrient sensing and SKN-1 activation

SKN-1 activity and regulation extend to changes in nutrient availability, redox status, and other environmental signals, highlighting its dynamic engagement with the cellular environment (Pang et al., 2014). Caloric restriction (CR) or dietary restriction (DR), characterized by a reduction in nutrient availability and/or metabolic energy without malnutrition, serves as a potent activator of SKN-1 (Onken and Driscoll, 2010). Dietary restriction has been established as a mechanism that extends the lifespan of *C. elegans*, conferring diverse health benefits. Notably, SKN-1 is one of the transcription factors involved in mediating this effect. In a study by Bishop and Guarente (2007), they showed that lifespan extension was nullified in *skn-1* mutants subjected to DR, and this attenuation was restored by introduction of a *skn-1* transgene (Cypser et al., 2013). Interestingly, the activation of *skn-1* proved necessary exclusively in the ASI neurons for DR-induced longevity. Expression of a *skn-1* transgene solely in the ASI neurons rescued the loss of DR-induced lifespan extension caused by *skn-1* deletion (Bishop and Guarente, 2007). This study suggests a neuroendocrine function for SKN-1, particularly in the ASI neurons, and points to a cell non-autonomous effect involving a systemic longevity signal downstream of SKN-1 (Bishop and Guarente, 2007; Sykiotis et al., 2011).

Further recognized as a longevity factor, SKN-1 is activated in various long-lived mutant backgrounds exhibiting altered metabolic homeostasis. SKN-1 stands as an indispensable contributor to the observed lifespan extension in these mutants, underscoring its significance in the regulation of aging processes (Bishop and Guarente, 2007; Tullet et al., 2008; Wang et al., 2010). SKN-1 is integrated into the insulin/IGF-1 signaling pathway, which plays a central role in nutrient sensing and metabolism. Specifically, the insulin/IGF-1 signaling (IIS) receptor DAF-2 plays a role in this regulatory cascade. Activation of DAF-2 triggers a downstream modulation of transcription factors, including the inhibition of SKN-1. Consequently, SKN-1 is hindered from regulating its target gene networks (Tullet et al., 2008; Onken and Driscoll, 2010). Experimental manipulations such as knockdown of DAF-2, either through mutation or RNA interference (RNAi), have been shown to activate SKN-1, resulting in long-lived and stress-resistant phenotypes in worms (Carroll et al., 1997). Importantly, SKN-1 is needed during adulthood to confer these beneficial effects (Onken and Driscoll, 2010; Grushko et al., 2021). The observed benefits of DR and decreased insulin signaling may be explained by a metabolic shift that optimally allocates available resources for the maintenance of overall fitness and viability (Sykiotis et al., 2011). Drawing parallels with worms, several studies reveal that reduced IGF-1 signaling exerts a comparable influence on lifespan extension in mice (Cohen et al., 2009; Vitale et al., 2019). Intriguingly, mutations in components of the IGF-1 pathway also correlate with longevity observed in humans (Vitale et al., 2019). Together, these findings indicate that the aging-regulating roles attributed to the IIS pathway are conserved from worms to mammals (Holzenberger et al., 2003; Willcox et al., 2008).

In recent investigations, microRNAs (miRNAs) have been identified to promote longevity, particularly under conditions of DR and reduced insulin signaling (Matai et al., 2023). miRNAs are short, non-coding RNAs that bind to complementary sequences in mRNA molecules, orchestrating the inhibition of protein translation or mRNA degradation, thereby crucially contributing to post-transcriptional regulation. A study by Matai *et al.* (2023) has shed light on the involvement of the *mir-229,-64,-65,-66* cluster of miRNAs in the context of DR and low-IIS scenarios. Intriguingly, this miRNA cluster is upregulated under DR and low-IIS scenarios, and in turn this cluster upregulates *skn-1* mRNA levels, revealing an interplay between miRNAs and SKN-1 (Matai et al., 2023). The interaction of the miRNA cluster (miR-229–66) together with SKN-1 introduces an indirect regulatory mechanism that transduces the effects of DR and low-IIS into lifespan extension. This regulatory cascade involves the modulation of several ubiquitin-mediated proteolysis and xenobiotic detoxification pathway genes (Matai et al., 2023). Understanding the molecular mechanisms by which SKN-1 responds to diet and metabolism in *C. elegans* can provide insights into the broader connections between nutrient sensing, oxidative stress response, and lifespan regulation in other organisms, including mammals.

### 2.4 Role of SKN-1 in lipid metabolism

Maintaining energy homeostasis is a fundament aspect of animal physiology, requiring intricate coordination in the metabolism of intracellular nutrients such as glucose, lipids, and amino acids (Pang et al., 2014). Animals employ sophisticated molecular mechanisms to ensure the continual fulfillment of organismal demands for growth, cellular maintenance, and reproduction (Efeyan et al., 2015; Lynn et al., 2015). SKN-1 has been implicated in the regulation of lipid metabolism, impacting both lipid storage and utilization in response to fluctuating nutrient availability (Pang et al., 2014; Lynn and Curran, 2015; Lynn et al., 2015). SKN-1 responds dynamically to changes in nutrient availability and can modulate its activity in response to shifting nutritional landscapes through interactions with signaling pathways, including the mitochondrial amino acid catabolism pathway, and insulin and IGF-1 signaling pathways (Tullet et al., 2008; Zarse et al., 2012; Pang et al., 2014). Recent studies propose that SKN-1-mediated transcriptional programs mechanistically link proline and fatty acid metabolism, providing a deeper understanding of the crosstalk between these pathways (Pang et al., 2014; Yen et al., 2020). Mutations in mitochondrial proline catabolism (*alh-6*) lead to mobilization of intestinal lipids during starvation, indicating a coupling with lipid metabolism in response to nutrient depletion (Pang and Curran, 2014). In these mutants, several lipid metabolism and fatty acid synthesis genes were upregulated during starvation, such as the fasting-induced lipase-1 (*flp-1*) and the fatty acid oxidation enzyme (*cpt-5*) (Pang and Curran, 2014). Notably, a loss-of-function mutation in *skn-1* abolishes the enhanced depletion of somatic lipid stores observed in *alh-6* mutants after fasting, indicating SKN-1’s role in mediating fasting metabolic responses (Pang and Curran, 2014).

Subsequently, gain-of-function mutations in *skn-1* (*lax188*) lead to a starvation-like status and can induce the expression of several metabolic genes; however, the full extent to which SKN-1 participates in organismal physiology and metabolism remains unknown (Paek et al., 2012). Work on *skn-1* gain-of-function mutant animals show these animals have an upregulation of FAO genes, such as *acs-1*, *cpt-4, cpt-6* (Pang et al., 2014)*,* as well as metabolism and starvation adaptation genes, such as *lips-3, atgp-2, acdh-6* (Paek et al., 2012)*.* Interestingly, these *skn-1gf* mutants exhibit the age-dependent somatic depletion of fat (Asdf) phenotype, characterized by the depletion of intestinal lipid stores without affecting germline lipid stores. This phenotype, associated with a starvation response dependent on vitellogenesis, which facilitates transport of stored lipids from the intestine to developing oocytes, emphasized the role of SKN-1 in the reallocation of lipids from soma to germline, potentially ensuring survival and fitness under challenging conditions (Lynn et al., 2015). This Asdf phenotype exhibits specificity to oxidative stress, as wildtype worms subjected to heat or osmotic stress failed to induce the observed lipid shift to the germline. Notably, treatment with *N-*acetylcysteine (NAC), a potent antioxidant, effectively suppressed the Asdf phenotype in *skn-1gf* mutants. This highlights the oxidative stress-dependent nature of the observed lipid distribution, emphasizing the central role of SKN-1 in coordinating the cellular response to oxidative challenges. Given its high expression in the intestine, SKN-1’s crucial role in responding to dietary changes becomes evident. The intestine, as a central tissue for nutrient absorption and metabolism, positions SKN-1 as a contributor to the overall metabolic homeostasis of *C. elegans,* underscoring SKN-1’s role in orchestrating lipid homeostasis and metabolic responses essential for the organism’s adaptation and survival.

Dietary lipids and lipid metabolism are critical for organismal adaptation to external conditions, lifespan regulation, and reproduction (Ma et al., 2020; Mejhert et al., 2022; Papsdorf et al., 2023). Lipids encompass a diverse array of hydrophobic molecules, including triglycerides, phospholipids, and steroids. The impact of lipids on longevity and the regulatory mechanisms of lipid metabolism during aging are subjects of ongoing investigation. Specific lipid profiles are associated with survival, with increased fat storage linked to aging, while certain lipids, such as mono-unsaturated fatty acids (MUFAs) like oleic acid and polyunsaturated fatty acids (PUFAs) like eicosapentaenoic acid (EPA), alpha-linolenic acid (ALA), and arachidonic acid (ARA), are implicated in regulating transcription factors like SKN-1 (Papsdorf and Brunet, 2019). During nutrient deprivation, stored lipids and amino acids are used instead of dietary glucose to satisfy organismal energy requirements. Lipids are mobilized as an energy resource through lipolysis and fatty acid oxidation (FAO) (Pang et al., 2014). Analysis of the *skn-1gf* animals revealed a reduction in oleic acid (C18:1), suggesting its deficiency as causative for the Asdf phenotype (Lynn et al., 2015). Knockdown of *fat-6* and *fat-7*, which encode enzymes converting stearic acid to oleic acid, phenocopied the Asdf phenotype (Lynn et al., 2015). Supplementation with biosynthetic precursors to oleic acid, including stearic acid, lauric acid, and alpha-linolenic acid, did not suppress Asdf in *skn-1gf* mutants. Investigations into lipid biosynthesis pathways in Lynn *et al.* (2015) indicated that under nutrient-poor and oxidative stress conditions, omega-3 and omega-6 PUFAs mediate the balance of lipid stores between the soma and germline, impacting reproduction and somatic aging (Lynn et al., 2015). Mutant animals unable to generate EPA or ARA exhibited the Asdf phenotype at a later time point than *skn-1gf* animals, highlighting the role of specific PUFAs in regulating this metabolic shift. The complexity of the relationship between lipid metabolism and SKN-1-mediated responses in maintaining cellular homeostasis is underscored by these studies. In a study by Qi *et al* (2017), the supplementation of ALA was found to extend lifespan in *C. elegans* through the regulation of the NHR-49 and SKN-1 transcription factors. Notably, ALA indirectly activated SKN-1 activation via oxidation of ALA to produce oxylipins, ultimately leading to the activation of SKN-1 and conferred longevity (Qi et al., 2017)*.* These studies emphasize the complexity between lipid metabolism and SKN-1-mediated responses in maintaining cellular homeostasis, with an emphasis on how specific lipids can influence and modulate the organism’s lifespan.

A study by Cedillo *et al.* (2023), elucidated the connection between SKN-1, biguanides, and ether lipid metabolism, highlighting their collective role in coordinating biguanide-mediated responses to dietary conditions and innate immune responses to promote longevity (Cedillo et al., 2023). Ether lipids, a diverse class of membrane lipids integral to cellular functions and homeostasis, contribute to membrane structure, stability, and fluidity, impacting crucial cellular processes such as membrane fusion and cellular signaling (Mejhert et al., 2022). Biguanides, including the widely prescribed metformin, are drugs commonly used to lower blood sugar levels by reducing glucose production in the liver. Metformin is often used to manage and treat type 2 diabetes, and is associated with various metabolic benefits, including weight control and improved insulin sensitivity (Martin-Montalvo et al., 2013). SKN-1 has been previously established as necessary for metformin-induced lifespan extension (Onken and Driscoll, 2010; Cedillo et al., 2023). This study further demonstrated that treatment with phenformin, a biguanide akin to metformin, induces the Asdf phenotype at day 3 of adulthood, similar to *skn-1gf* mutants (Lynn et al., 2015; Nhan et al., 2019). Notably, loss-of-function mutations in ether lipid biosynthetic genes thwarted the phenformin-induced Asdf phenotype, suggesting a mechanistic link between ether lipids and *skn-1*-dependent metabolic defenses (Cedillo et al., 2023). Moreover, loss-of-function *skn-1* animals nullified the Asdf phenotype associated with phenformin treatment, indicating the essential role of SKN-1 in this biguanide-mediated Asdf lipid shift. Phenformin treatment induced expression of the innate immune response gene *dod-24* in a SKN-1-dependent manner. Collectively, this study indicates that biguanides, specifically phenformin, can extend the lifespan by activating metabolic stress defenses through ether lipids, with SKN-1 playing a pivotal role. Although the precise molecular mechanisms underlying metformin’s interaction with SKN-1 are an area of ongoing research, these studies offer valuable insights into the potential involvement of Nrf2 in mediating the health-promoting effects of biguanides, with implications for understanding the broader impacts of metformin on cellular health and longevity in higher organisms, including humans.

## 3 SKN-1 responses to pathogens

Transitioning from the influence of specific lipids on SKN-1-mediated responses, an exploration into the transcripts regulated by SKN-1 that contribute to the Asdf phenotype unveils a deeper understanding of the molecular mechanisms involved. Previous findings revealed that SKN-1 triggers a metabolic stress defense response, facilitating somatic lipid depletion by enhancing lipid utilization and promoting innate immunity gene expression, counteracting canonical oxidative stress responses (Nhan et al., 2019). Lynn *et al.* (2019) demonstrated that manipulating methylation states, specifically H3K4me3, Histone H3 at lysine 4, through knockdown of *wdr-5* or *rbbp-5,* or with studies on *wdr-5lf;skn-1gf* double mutants, reversed the Asdf phenotype in *skn-1(lax188)* gain-of-function mutant animals, restoring somatic lipids and extending lifespan (Nhan et al., 2019). This study highlights the role of H3K4me3 trimethylation in fine-tuning deregulated SKN-1 transcriptional activity away from innate immunity targets, mitigating the negative metabolic outcomes. Furthermore, exposure to subliminal paraquat-induced oxidative stress redirected *skn-*1*gf* activity from pathogen response genes, simultaneously restoring somatic lipid distribution. Transcriptional analyses of this paraquat exposure unveiled reduced *skn-1gf* association with the promoter regions of innate immunity genes, *dod-24* and *endu-2*, and enhanced association with the oxidative stress gene, *gst-4*. This study opened a novel avenue for activated SKN-1 transcription factor studies, emphasizing the critical role of immune activation in coordinating pathogen responses and adaptive adjustments.

The use of bacteria as the primary nutritional source for *C. elegans* serves as a valuable approach to investigate host-pathogen interactions. The immune defense of *C. elegans* against pathogenic stress involves a sophisticated network of molecular pathways, with SKN-1 emerging as a master regulator (Papp et al., 2012a; Liu et al., 2022). In response to pathogenic encounters, SKN-1 orchestrates a range of responses to maintain host homeostasis, ensuring survival and contribute to longevity. In adult *skn-1gf* mutants exhibiting the Asdf phenotype, transcriptional and Gene Ontology analyses revealed a strong enrichment of immune and pathogen response genes, including *dod-24, endu-2,* and *clec-66,* suggesting that SKN-1 plays a crucial role in regulating innate immune response genes during later stages of development (Nhan et al., 2019). Interestingly, a *nsy-1gf* mutant, which constitutively activates the immune response via the p38 mitogen-activated protein kinase (MAPK) PMK-1 pathway, revealed a similarly Asdf phenotype. The suppression of this phenotype in the *skn-1* (*zu135*) loss-of-function mutant points to the essential role of SKN-1 activity for the NSY-1/PMK-1 pathways and the subsequent transcription of innate immunity target genes (Nhan et al., 2019). While these studies were conducted on the non-pathogenic OP50 diet, other studies utilized the opportunistic human pathogen *Pseudomonas aeruginosa* (PA14) strain, known to infect and ultimately kill nematodes (Cezairliyan et al., 2013; Pukkila-Worley, 2016). In the context of PA14 infection, SKN-1 was identified as a regulator of pathogen resistance, and this activation was dependent on the TIR-1/PMK-1 signaling pathway (Papp et al., 2012b). By dissecting these pathogen-specific interactions, a deeper understanding of the adaptability and specificity of SKN-1 in response to diverse metabolic threats is achieved. These studies, ranging from SKN-1 activation pathways to its contributions to immune effectors and stress resilience, can unravel additional layers of complexity in host-pathogen dynamics.

## 4 Mechanisms of molecular regulation of SKN-1

### 4.1 Inhibitory regulation of SKN-1 via the ubiquitin proteasome

**FIGURE 2:**
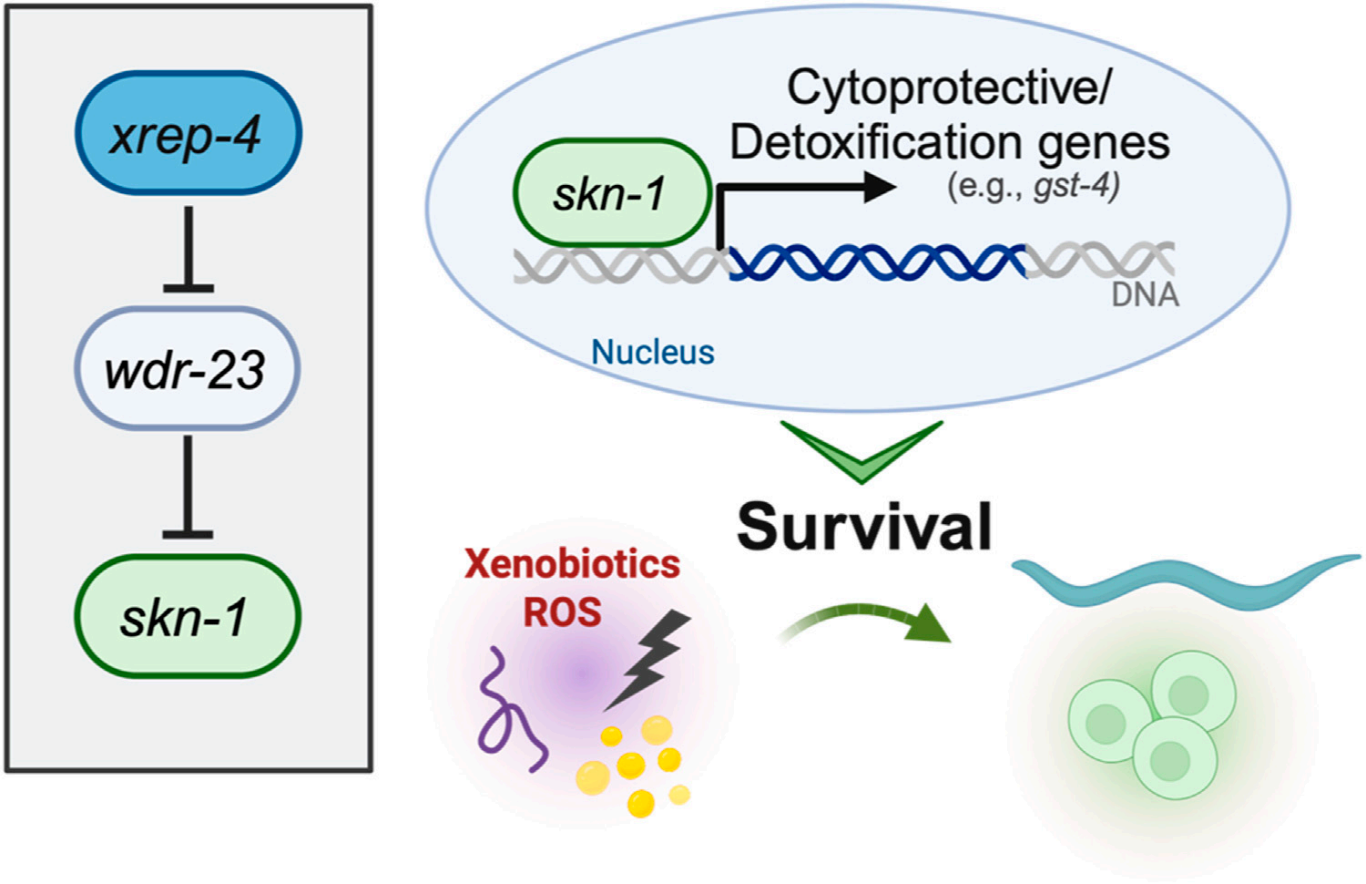
Genetic regulators of SKN-1 activity.

Like Nrf2, proteostatic turnover is the primary way SKN-1 is regulated in *C. elegans* (Figure 2) (Choe et al., 2009; Taguchi et al., 2011; Tang and Choe, 2015). Worms do not have a Keap1 orthologue, however SKN-1 associates with the WD Repeat Protein 23 (WDR-23) which is an E3 ubiquitin ligase substrate adaptor (Choe et al., 2009). WDR-23 will associate with CULlin-4 (CUL-4) allowing for the polyubiquitylation of SKN-1 and subsequent degradation by the proteasome (Choe et al., 2009). RNAi knockdown of *wdr-23* will result in the accumulation of SKN-1 in the intestinal nuclei (Choe et al., 2009). However, *wdr-23* has two isoforms, *wdr-23a* and *wdr-23b*, each having a distinct subcellular localization, WDR-23A localizing to mitochondria and WDR-23B localized to the nucleus (Staab et al., 2013). Rescue constructs that restore only WDR-23b in *wdr-23* loss-of-function mutants display normal SKN-1 activity in SKN-1 activity reporters, while rescues restoring only WDR-23a do not (Spatola et al., 2019). Furthermore, knockdown of *wdr-23* resulted in a synergistic activation of SKN-1 which indicate the mechanisms that control SKN-1 functions expand beyond proteostatic levels of SKN-1 abundance (Paek et al., 2012). This presents an interesting model for WDR-23 regulation of SKN-1 where SKN-1 is turned over by nuclear WDR-23b while cytoplasmic WDR-23 associates at the mitochondria and binds mitochondrial SKN-1 and acts a sensor for mitochondrial stress and ROS accumulation (Spatola et al., 2019). This model would perfectly mirror the mammalian model for Nrf2 regulation via Keap1; however, to date there is no evidence that the cystine residues on WDR-23 are oxidized by electrophilic compounds. Furthermore, mammals have a conserved orthologue of WDR23 which has been shown to regulate Nrf2 independently of KEAP1 (Lo et al., 2017), this suggests that mammalian regulation is diverged from *C. elegans* and that insights into WDR-23 in the worm may yield information on how WDR23 functions in mammals.

Regulation of SKN-1 by WDR-23/CUL-4 and the canonical ubiquitin proteasome has been established, however proteostasis is also mediated via two other ubiquitin-like modifying pathways (Enchev et al., 2015; Vertegaal, 2022). These include the SUMOylation pathway and NEDDylation pathway (Enchev et al., 2015; Vertegaal, 2022). Both pathways mediate the addition of small ubiquitin like moieties to proteins, chiefly to signal degradation; however, both pathways are involved in other processes such as signaling, cell cycle, DNA damage response, and immunity (Enchev et al., 2015; Vertegaal, 2022). The constitutively active *skn-1* allele, *lax188*, contains a glutamic acid to lysine substitution (Paek et al., 2012). This mutant displays a trade-off between increased stress tolerance and decreased longevity (Paek et al., 2012; Nhan et al., 2019; Turner et al., 2023). Using RNAi to knockdown both the SUMOylation and NEDDylation enzymes in the worm, the activated SKN-1 allele was shown to be more sensitive to loss of NEDDylation, accumulating in the intestinal nuclei, while wildtype SKN-1 remained excluded (Turner et al., 2023). Loss of SUMOylation had no effect on the wildtype or constitutively active SKN-1 (Turner et al., 2023). These results have given new insight into the regulation of SKN-1. Given that SUMOylation did not have an effect on the localization of SKN-1, it is likely that SKN-1 is a specific substrate of NED8. There is much left to understand about NEDDylation on SKN-1, whether it is mono- or poly-NEDDylated and if it has chains of NEDDylation. It is also unknown if SKN-1 is broadly NEDDylated or if specific conditions are required for it to be NEDDylated. Investigations into the proteostatic turnover of SKN-1 have yielded insights into the intricate nature of SKN-1 regulation via the ubiquitin proteasome. Additional studies are needed to determine the sites of ubiquitylation and NEDDylation on SKN-1, these may also reveal if SKN-1 is poly- or mono-ubiquitinylated/NEDDylated. *In vivo* identification of the subcellular localization of SKN-1C and SKN-1B isoforms will be crucial to understand where SKN-1 is sequestered in the cell and how it may evade degradation as a form of signaling.

### 4.2 Inhibitory regulation by kinases

SKN-1 regulation outside of proteasomal degradation is mediated by post-translational phosphorylation modifications (Blackwell et al., 2015). Among the various kinases that regulate SKN-1, one of the more defined is GSK-3, a kinase first discovered to control the synthesis of glycogen (An et al., 2005). Interestingly, GSK-3 is another developmentally required protein (Maduro et al., 2001a). In the embryo, it controls the establishment of C blastomere fate that will develop into the epidermis, it does so by inhibiting the transcriptional activity of SKN-1 in the P_2_ blastomere (Maduro et al., 2001b). Post-embryonically, GSK-3 phosphorylates SKN-1 at serine residues S393 and S432 (An et al., 2005). This phosphorylation prevents the intestinal accumulation of SKN-1, with both post-embryonic RNAi to *gsk-3* as well as serine to alanine mutations at GSK-3 phosphorylation sites, resulting in the nuclear accumulation of SKN-1 in intestinal nuclei (An et al., 2005). Another inhibitory phosphorylation event is mediated via AKT kinase. Insulin-like signaling in *C. elegans* is mediated through DAF-2 activation of AKT (Tullet et al., 2008; Ewald et al., 2015). AKT inhibits SKN-1 by phosphorylating serine residue S12, preventing intestinal accumulation (Tullet et al., 2008; Ewald et al., 2015). Reduction of Insulin-like signaling results in the accumulation of SKN-1 (Tullet et al., 2008; Blackwell et al., 2015). Regulation of SKN-1 via insulin-like signaling places SKN-1 in longevity promoting pathways in parallel to those mediated via DAF-16 (Tullet et al., 2008; Ewald et al., 2015), further connecting SKN-1 to mechanisms controlling metabolic homeostasis.

SKN-1 is also regulated by the mTORC-2 pathway (Jones et al., 2009; Robida-Stubbs et al., 2012; Ruf et al., 2013; Mizunuma et al., 2014). mTORC-2 subunit RICT-1 is responsible for phosphorylating and activating kinase SGK-1 (Jones et al., 2009). A particular serine residue has yet to be determined for SGK-1, however western blots for phosphorylated SKN-1 show that SGK-1 phosphorylates SKN-1 in the C-terminal region 300-623aa (Jones et al., 2009). Like other inhibitory kinase systems, loss of either *rict-1* or *sgk-1* results in the nuclear accumulation of SKN-1 in the intestinal nuclei (Jones et al., 2009; Robida-Stubbs et al., 2012; Ruf et al., 2013; Mizunuma et al., 2014). Phosphorylation of SKN-1 by the mTORC-2 pathway implicates SKN-1 in nutrient sensing and growth regulation. Previously it was shown that feeding two different bacterial diets had opposing effects on lifespan under inhibited mTORC-2 conditions (Mizunuma et al., 2014). Determining the bacterial or metabolic signals that cause differential effects on lifespan as well as the tissue-specificity will be key in understanding mTORC-2-mediated SKN-1 longevity.

### 4.3 Positive regulation by the MAPK pathway

Kinases can also positively regulate SKN-1. The p38 mitogen-activated protein kinase (MAPK) pathways are evolutionarily conserved kinase cascades that are required for responses to various stressors including oxidative stress and pathogenic infection (Kurz and Tan, 2004; Inoue et al., 2005; Hoeven et al., 2011). Canonical MAPK members in *C. elegans* include NSY-1,SEK-1, and PMK-1 (Kurz and Tan, 2004; Inoue et al., 2005; Hoeven et al., 2011). Genetic ablation of any of these results in an inability of intestinal SKN-1 to accumulate and respond to stress (Inoue et al., 2005). The terminal kinase PMK-1 phosphorylates SKN-1 at two known serine residues, S164 and S430, accordingly genetic ablation of these serine residues to alanine ablates the ability of SKN-1 to accumulate into the intestinal nuclei (Inoue et al., 2005). The p38 MAPK network is nearly universally required for stress adaptation in the intestine of the worm, however the importance of the p38 MAPK pathway and Nrf2 activation is less understood (Kurz and Tan, 2004; Inoue et al., 2005; Sun et al., 2009; Hoeven et al., 2011). While phosphorylation via the MAPK pathway is required for response to most biotic stimuli, it is unclear if constitutively active SKN-1 is continually phosphorylated by the MAPK pathway or if it circumvents it entirely. Studies on the phosphorylation of activated SKN-1 mutants will provide insight into the regulation of activated SKN-1. Furthermore, PMK-1 phosphorylation sites are shared between all SKN-1 isoforms, and it is currently unknown if there is any selectivity of the MAPK pathway to phosphorylate certain SKN-1 isoforms over others, and given the proposed specific roles each isoform plays, investigating this may provide insight into SKN-1 target selectivity.

### 4.4 Additional regulation by post-translational modifications

**FIGURE 3:**
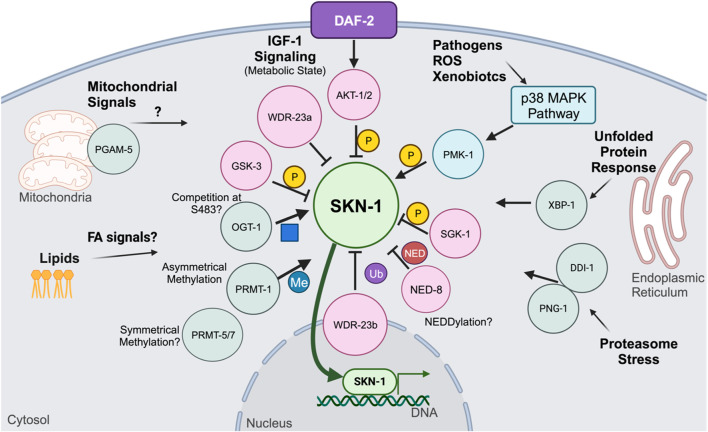
Post-translation modifications and signaling pathways that mechanistically affect SKN-1 activity.

A bevy of other post-translational modifications regulate SKN-1 activity (Figure 3). Recently, SKN-1 was shown to be regulated via *O*-linked N-acetylglucosamine (*O*-GlcNAc) cycling. Mutants for *ogt-1*, the transferase responsible for the addition of *O*-GlcNAc show impaired response to oxidative stress and a reduced SKN-1 accumulation in the intestinal nuclei (Li et al., 2017). Accordingly, mutants for *oga-1*, the acetylase responsible for removing the modification, have elevated SKN-1 target gene expression and increased SKN-1 intestinal accumulation (Li et al., 2017). Further biochemical analysis demonstrated that SKN-1 is *O*-GlcNAcylated at serine residue S470 and threonine residue T493 which neighbor the GSK-3 phosphorylation site and that during stress conditions, the GSK-3 site was less phosphorylated and the OGT-1-targeted residues were *O*-GlcNAcylated (Li et al., 2017). This analysis proposes an interesting competition dynamic between GSK-3 inhibitory phosphorylation and activating *O*-GlcNAcylation (Li et al., 2017). *O*-GlcNAcylation on mammalian Nrf2 has yet to be deeply understood, however genetic analysis of Ogt in mammals is difficult since genetic ablations of *O*-GlcNAc cycling enzymes is lethal (Li et al., 2017; Levine et al., 2021).

Another post-translational modification shown to regulate SKN-1 activity is arginine methylation (Li et al., 2019). SKN-1 has been shown to be asymmetrically methylated by PRMT-1 at arginine residues 484 and 516 (Li et al., 2019). During oxidative stress, PRMT-1 binds to SKN-1 and methylates its target arginine residues, which results in SKN-1 activation (Li et al., 2019). Loss of PRMT-1, either through RNAi knockdown or genetic mutation, results in the inability for SKN-1 to accumulate in the intestinal nuclei and a reduction in the expression of phase II detoxification genes (Li et al., 2019). Loss of the Arginine residues where SKN-1 is methylated did not alter its nuclear accumulation like total loss of PRMT-1, suggesting that phosphorylation contributes significantly to the nuclear accumulation and arginine methylation may play a role in DNA binding enhancement (Li et al., 2019). SKN-1 contains several other Arginine residues that may be methylated, further proteomic analysis may reveal additional methylation on SKN-1 (Li et al., 2019). Furthermore, while PRMT-1 catalyzes asymmetrical methylation, it is unknown if any symmetrical methylation occurs on SKN-1 and how it may affect DNA binding dynamics. Further analysis of arginine methylation may yield new insights into how SKN-1 targets are specified and selected, and future work should focus on the methylation status of different SKN-1 isoforms as well as the methylation status under various stressors, such as pathogenic and metabolic.

Additional RNAi-based screens of kinases in *C. elegans* have revealed additional regulatory kinases that function to inhibit SKN-1 activity. Kinases from this screen include *nekl-2*, *ikke-1*, and *mkk-4* (Kell et al., 2007). Each of these kinases are from disparate biological processes which include the cell cycle and glucose metabolism, however no biochemical analyses have been done to determine if such kinases interact directly with SKN-1 or if these kinases regulate processes that modulate SKN-1 downstream (Kell et al., 2007). A determination of whether SKN-1 is a direct target of each of these kinases and comprehensive identification of the residues modified is critical to understanding the mechanistic roles these PTMs play in controlling SKN-1 activity.

## 5 Modulators of SKN-1 activity

### 5.1 Molecular triggers of SKN-1 activity

The activation of SKN-1 in *C. elegans* represents a complex regulatory network crucial for its adaptive responses to environmental cues. SKN-1 activity is finely tuned by a spectrum of stimuli, with oxidative stress serving as a primary inducer. Positive regulators, including phosphorylation by p38 MAPK PMK-1, and the ubiquitin ligase adaptor protein SKR-1*,* activate SKN-1 in response to ROS (Troemel et al., 2006; Wu et al., 2016). Additionally, the acetyltransferase enzyme CBP-1, a transcriptional regulator in cellular processes such as oxidative stress resistance and aging, has been shown to directly modulate SKN-1 translocation to the nucleus. In response to stress, CBP-1 promotes SKN-1-dependent transcription of protective genes (Ganner et al., 2019). Knockdown of *cbp-1* suppressed the expression of said SKN-1 target genes, indicating that CBP-1 drives SKN-1 transcription. Conversely, negative regulators such as serum and glucocorticoid-inducible kinase-1 (SGK-1) and WDR-23 inhibit SKN-1 nuclear accumulation and target it for degradation, respectively (Tullet et al., 2008; Choe et al., 2009). Additional positive regulators have been studied, including the F-box protein XREP-4, which functions with SKR-1 to negatively inhibit the activity of WDR-23 (Fukushige et al., 2017; Wu et al., 2017). Recently, we have defined a novel gain-of-function mutation in the *xrep-4* locus, which contributes to constitutive activation of wildtype SKN-1 (Ramos and Curran, 2023). In established genetic models of constitutive SKN-1 activation, two paradigms include loss-of-function alleles of *wdr-23* and gain-of-function alleles of *skn-1.* Both hinder the ubiquitin proteasome-mediated turnover of SKN-1, leading the sustained activation (Paek et al., 2012; Spatola et al., 2019). *wdr-23lf* mutants exhibit enhanced resistance to oxidative stress, accompanied by an upregulation of stress response, metabolic, and signaling genes (Staab et al., 2013; Tang and Choe, 2015). *skn-1gf* mutants, as discussed earlier, undergo a transcriptional redirection that influences altered survival responses (Nhan et al., 2019). Extending this paradigm, *xrep-4gf* mutants also show increased expression of genes associated with glutathione transferase activity, immune system responses, and xenobiotic responses. Intriguingly, there is an overlap of 1796 genes with *skn-1gf* mutants, indicating shared molecular signatures. Despite exhibiting a similar premature age-related decline as *skn-1gf* animals, the phenotypic impact of *xrep-4gf* animals is comparatively less severe in terms of both healthspan and lifespan, emphasizing nuances in the outcomes of SKN-1 activation through distinct genetic models (Ramos and Curran, 2023).

In addition to genetic regulators, SKN-1-mediated transcriptional activation through disruption of amino acid catabolism or exogenous amino acid supplementation have also been elucidated. Perturbation of amino acid catabolic pathways, such as proline catabolism, leads to a buildup of toxic catabolites, triggering SKN-1-related transcription through ROS accumulation (Pang et al., 2014; Yen et al., 2020). Similar SKN-1 transcriptional responses have been reported with perturbations of tryptophan, threonine, and most recently, histidine catabolism (Frankino et al., 2022). In a study by Frankino *et al.* (2022), knockdown of the conserved amidohydrolase T12A2.1/*amdh-1* results in the accumulation of the catabolite 4-imidazolone-5-propanoate, serving as a mediator for the activation of a specific subset of SKN-1-regulated genes. Interestingly, this activation is independent of the P38-MAPK pathway but involves the nuclear factor ELT-3, nuclear hormone receptor NHR-49, and mediator complex subunit MDT15. Most recently, Wu *et al.* (2023) proposed a cascade of events activating SKN-1 initiated by alpha-ketobutyrate (α-KB) supplementation (Wu et al., 2023). Addition of α-KB leads to increased expression of the enzyme ACOX1, which is involved in the peroxisomal fatty acid β-oxidation pathway. This process generates hydrogen peroxide (H_2_O_2_), activating SKN-1, and subsequently promoting the translocation of SKN-1 to the nucleus to upregulate genes involved in cellular defense against oxidative stress (Wu et al., 2023). These findings highlight the multifaceted regulator mechanisms modulating SKN-1 activity, in addition to the intersection between metabolism and stress response pathways in orchestrating cellular homeostasis.

The intricate regulatory mechanisms uncovered in *C. elegans’* SKN-1 have parallels in mammalian systems, particularly Nrf2, where constitutive activation is implicated in cancer progression and chemoresistance (Sykiotis and Bohmann, 2010; Wu et al., 2019). Understanding these regulatory networks not only sheds light on fundamental cell biology questions but also holds clinical relevance, offering insights into diseases characterized by dysregulated transcription factors like Nrf2. Such findings could have implications in various fields, including pharmacology, as they suggest potential therapeutic avenues for modulating oxidative stress-related pathways. Understanding these molecular triggers not only provides insights into the nuanced regulatory mechanisms governing SKN-1 but also unravels the interconnected signaling pathways that contribute to the adaptive responses to changing environment conditions.

### 5.2 Insights into SKN-1 regulatory control

The importance of precisely regulating SKN-1/NRF2 activity becomes evident when considering the repercussions of chronic activation. Despite the crucial role of SKN-1 in mounting a protective response to various stressors, animals with sustained or constitutive activation of SKN-1 experience detrimental outcomes, highlighting the essentiality of regulating this cytoprotective pathway (Pang et al., 2014; Blackwell et al., 2015; Nhan et al., 2019). Studies on constitutively active SKN-1 mutants, such as *skn-1gf* and *xrep-4gf,* reveal age-dependent phenotypes, initially presenting enhanced stress resistance early in life but eventually exhibiting the Asdf phenotype, prioritizing reproductive fitness over somatic health (Lynn et al., 2015). This dysregulation of cellular lipid homeostasis contributes to diminished health and alters the organism’s overall lifespan (Pang et al., 2014; Lynn et al., 2015; Nhan et al., 2019). The capacity of epigenetic modifications to reshape the regulation of crucial physiological processes by influencing chromatin association underscores an exciting avenue for research. Chromatin modifications, including alterations in DNA packaging, emerge as pivotal, long-lasting mechanisms governing gene expression, particularly those implicated in cellular maintenance and longevity (Papsdorf and Brunet, 2019). Studies of the transcriptional redirection of constitutively active SKN-1 in *skn-1gf* mutants unveil the remarkable ability to fine-tune protective responses, adapting them to the specific requirements for reinstating homeostatic balance (Nhan et al., 2019). Of significance is the finding that activated SKN-1 transcription can be intentionally redirected, offering a potentially adaptable strategy to address diseases marked by aberrant transcriptional regulation. In the context of *skn-1gf* mutants, an RNAi screen targeting transcriptional regulators identified mediator subunit MDT-15, a lipid metabolism-regulating transcription factor, as a suppressor of constitutive SKN-1 activation (Pang et al., 2014). This study suggests that MDT-15 interacts with SKN-1 in the intestine, coordinating responses to different dietary conditions, metabolic regulation, and oxidative stress (Pang et al., 2014; Hu et al., 2018). Recent findings have identified another suppressor, a gain-of-function mutation in the dicer-related helicase gene, *drh-1,* which temporarily delays the adverse effects of SKN-1 activation (Turner et al., 2023). DRH-1, known for detecting viral double-stranded RNA (dsRNA) and regulating transcription of immune response genes upon viral infection, works to oppose the increased transcriptional load resulting from *skn-1gf,* highlighting the intricate balance required for maintaining cellular homeostasis (Sowa et al., 2020; Turner et al., 2023). However, the temporary relief provided by DRH-1 activation (*drh-1gf*) emphasized the critical importance of turning off cytoprotection to ensure proper physiological outcomes and prevent the adverse consequences stemming from chronic SKN-1 activation. Together, these findings hold promise for innovative therapeutic strategies aimed at correcting dysregulated molecular pathways to improve overall health.

## 6 Concluding remarks and perspectives

### 6.1 Mechanistic questions about SKN-1 biology and implications for human aging

Regulation of SKN-1 activity is incredibly complex. Foundational studies have given solid framework to our understanding of the mechanisms that govern transcriptional activation. However, to achieve a deeper understanding of SKN-1 biology, tissue, cell, and even sub-cellular investigations on the function of SKN-1 will need to be done. Understanding how SKN-1 interacts in each tissue will be paramount to further understanding tissue-specific roles that can be paralleled to mammalian tissues. Additionally, we do not understand what signaling molecules are used to communicate between tissues. While specific targets of each isoform of SKN-1 have been proposed, understanding how each SKN-1 isoform synergizes with the others is a key gap in knowledge. Continuing to study SKN-1 and metabolism will be paramount as healthy diets become therapeutic strategies for aging human populations. Using constitutively active SKN-1/Nrf models will also help fill the major knowledge gap about the deactivation of SKN-1/Nrf. Considering that hyperactive Nrf2 is a cancer hallmark in some tissues (Kansanen et al., 2013), it is paramount to understand how SKN-1/Nrf2 can be turned off or redirected once activated. The finding that activated SKN-1 can have its transcriptional profile redirected by exposure to different acute stresses has implications for the control of SKN-1 in constitutively active pathologies, such as cancer. SKN-1 is regulated by many post-translational modifications where some, like arginine methylation, contribute to target specificity and not SKN-1 function as a whole. Thus, it is possible that pharmacological targeting of SKN-1 post-translational modification could fine tune SKN-1/Nrf2 activity. Continual study of the intricate regulatory networks of SKN-1/Nrf2 may help identify future therapeutics to allow for beneficial activation of SKN-1/Nrf2 and the suppression of runaway activation in human pathologies.
